# Genomic Data Governance Challenges Between Research and Clinical Care: A Scoping Review

**DOI:** 10.1111/hex.70889

**Published:** 2026-10-03

**Authors:** Alice Kim, Bronwen Merner, Mark Taylor, Yingyi Luo, Fabian Cannizzo, Alan Petersen, Clara Gaff, Ainsley J. Newson, Kate Marchant, Louse Keogh

**Affiliations:** ^1^ Centre for Health Equity, Melbourne School of Population and Global Health University of Melbourne Melbourne Australia; ^2^ Melbourne Law School, University of Melbourne Melbourne Australia; ^3^ Faculty of Arts, Monash University Melbourne Australia; ^4^ National Centre for Indigenous Genomics, Australian National University Canberra Australia; ^5^ Department of Paediatrics Melbourne Medical School, University of Melbourne Melbourne Australia; ^6^ Murdoch Children's Research Institute Melbourne Australia; ^7^ Melbourne Genomics Health Alliance, Walter and Eliza Hall Institute for Medical Research Melbourne Australia; ^8^ Sydney Health Ethics, Sydney School of Public Health, Faculty of Medicine and Health, University of Sydney Sydney Australia; ^9^ LINEAGE GUARD Community Advisory Group Melbourne Australia

**Keywords:** community involvement, data governance, ELSI, genomic data, health equity, informed consent, management, translational sciences

## Abstract

**Introduction:**

The translational nature of genomics highlights the importance of developing a robust genomic data governance framework. To inform the development of such a framework for Australia, we conducted a scoping review to identify genomic data governance and management differences between research and clinical care.

**Methods:**

For this scoping review, four databases were searched and 9528 relevant articles published between January 2013 and October 2024 were identified. After screening, 43 empirical, legal and theoretical/commentary articles were included. The differences reported to affect genomic data governance and management practices across clinical and research settings were extracted and synthesised.

**Results:**

Fifteen empirical articles revealed that the ‘community’ and the ‘workforce’ reported different consent priorities and differing understandings of the expected benefits of providing genomic data for research. Three legal articles reiterated the influence of regulatory regimes on genomic data practices and rights. Lastly, all twenty‐five theoretical/commentary articles were authored by researchers; none by community representatives. While 3/25 articles described the genomic data practices of rare and undiagnosed disease patient populations, 22/25 examined the ethical, legal and social implications arising from genomic data. Among the latter, 14/22 proposed ‘solutions’ to address identified genomic data challenges associated with consent, returning research genomic data to patients and advancing precision medicine.

**Conclusion:**

Differences in understanding, expectations and regulatory regimes can challenge genomic data governance and management practices and perceptions. Considering the implications of these challenges, meaningful community involvement will be critical in developing a governance framework that responsibly leverages genomic data to deliver more equitable health care.

**Lived Experience or Public Contribution:**

Members of the Genetic, Undiagnosed and Rare Disease (GUARD) Collaborative – the LINEAGE Consortium's Community Advisory Group partner – Emma Bonser, Monica Ferrie, Alex Fulton, Caroline Kiefer, Kate Marchant, Tony Neilson, Maya Pinn, Tania Prior‐Smith, Heather Renton, Susie Roczo‐Farkas and Joel Taggart contributed community perspectives and critical reflections during discussions of early findings, informing the development of this manuscript. Kate Marchant, a LINEAGE‐GUARD member, is an author of this manuscript, contributing critical revisions and approving the final manuscript.

## 1 Introduction

Efforts to realise genomic medicine in routine health care, and precision medicine more broadly, are challenging the historical dichotomy between research and clinical care. The key goals of clinical care and research have historically been different; the former aims to provide direct benefit to the patient being treated while the latter is designed to advance generalisable knowledge that can benefit society [1]. Because they have traditionally been considered as separate enterprises, research and clinical care are governed by their own frameworks, regulations and practices. However, the increasing use and relevance of genomics and the data this technology generates are one example of how this boundary is beginning to ‘blur’ [1, 2], particularly against a background of broader moves to learning healthcare systems [3]. As such, it is imperative to understand the ethical, legal and social implications and challenges arising from the existing differences between clinical and research settings to ensure genomic data are appropriately governed and managed across these settings.

One venture highlighting the implications and challenges of the blurring of research and clinical boundaries is translational genomics research. Here, eligible patients, often recruited in healthcare settings, provide consent for their genomic data to be used for research purposes. This can challenge the research‐clinical boundary as different genomic data actors – namely patients, participants, and workforce such as researchers and clinicians – may hold different expectations and understandings of why and how genomic data are used. This may be further complicated if a genomic data actor is engaged across both ventures — for example, a patient is also a research participant. Moreover, the translational nature of genomics can obscure which activities are clinical or research. These factors illustrate the importance of addressing the governance, management and practice challenges that arise when clinical and research activities co‐occur, to ensure transparent and ethical engagement with genomic data [2, 3, 4, 5, 6]. Thus, the implications of the enduring research‐clinical boundary need to be considered in establishing genomic data governance.

Addressing this challenge is a goal of the Global Alliance for Genomics and Health (GA4GH). Established in 2013, GA4GH aims to enable responsible sharing of clinical and genomic data across research and health care by developing interoperable standards, policies and frameworks [7]. Through its international programme, GA4GH aims to ensure all populations can access the benefits of genomic medicine [7].

Many countries are working to implement genomics into their healthcare systems [8, 9, 10]. In Australia, Federal and state/territory governments have invested significantly in genomics research and implementation activities. One investment is the LINEAGE Consortium (‘LINEAGE’). Integrating expertise from bioethics, law, social science and lived experience, LINEAGE aims to develop an ‘*empirically‐informed … and contextually‐appropriate framework for the ethical governance of genomic data*’ [11]. Building upon the Blueprint for a National Approach to Genomic Information Management (the ‘NAGIM Blueprint’) [12], LINEAGE's work aims to address the question: How should genomic datasets be governed effectively and responsibly?

To inform this work, we conducted a scoping review of peer‐reviewed literature to address the question: How do the differences between clinical and research settings challenge genomic data governance and management? To our knowledge, no such review has been undertaken. In doing so, this review contributes understanding on the implications of these differences, providing important considerations in developing evidence‐informed genomic data governance.

## 2 Methods

This study was guided by the pragmatic paradigm, a theoretical framework which prioritises methods and decisions that best generate actionable knowledge [13]. Reflecting the pragmatic paradigm, a scoping review was chosen to address our research question as it provides a rigorous approach for synthesising the breadth of available evidence on a specific topic across defined contexts, identifying key concepts, methods and gaps, rather than a narrow focus to answer a tightly defined research question [14]. This review's protocol was informed by Arksey and O'Malley's guidance on scoping reviews [15]. Its five stages – identifying the research question; identifying relevant studies; study selection; charting the data; and collating, summarising and reporting the results ‐ were used to structure our methods. This protocol was registered to the Open Science Framework on 3 October 2024 [16].

### 2.1 Stages 1 and 2: Identifying the Research Question and Relevant Studies

As recommended by the Joanna Briggs Institute (JBI), the Participants, Concept and Context (PCC) framework [17] was used to define the scope of this review, and the development of the search strategy and selection criteria to ensure it addressed the review's aims (refer to Table 1). This involved mapping the components of the research question to the most suitable participants (any actor of human genomic/genetic data), concept (any element of human genomic/genetic data governance and/or management) and context (both clinical and research settings). Decisions regarding the databases and search strategies (refer to Supplementary material 1), developed in consultation with an Expert Librarian, aimed to provide a broad and balanced coverage of the topic by drawing on diverse disciplines and sources while ensuring the search strategy was systematic and high‐quality. For pragmatic reasons, searches were limited to English language articles only. Publications from January 2013 were included to reflect the advent of next‐generation sequencing and the formation of bodies such as the GA4GH [7]. We searched four databases — Medline, Embase, Web of Science and Scopus — in October 2024.

**Table 1:** Review scope and article screening selection criteria mapped to the participants, concept and context framework.

	Criteria
Participants	Any actor of human genomic or genetic data.
Concept	The article discusses any element of human genomic or genetic data governance and/or management.
Context	The article discusses human genomic data governance and/or management in both clinical and research settings.
Publication language	English language
Study type	Empirical qualitative, quantitative, and mixed methods studies (including description of data collection and analysis methods) Opinion pieces, perspectives, commentaries Other analysis – policy, legal, normative ethical, …

### 2.2 Stages 3 and 4: Study Selection and Charting the Data

All search records were imported into the review management platform Covidence [18]. After duplicate records were removed, the title/abstract of each record was screened by AK using the outlined selection criteria (Table 1). Full‐texts for included records were then retrieved and independently screened by two authors. During full‐text screening, two authors (AK and BM) iteratively refined the selection criteria to only include articles that specifically addressed the review's aim (refer to Supplementary material 2 for the comprehensive criteria). All conflicts were resolved through discussion.

### 2.3 Stage 5: Collating, Summarising and Reporting the Results

A data extraction matrix was developed to capture data relevant to the research question. Extracted data were then analysed using framework analysis [19], involving five stages: familiarisation, identifying a thematic framework, indexing, charting, and then mapping and interpretation [19]. Familiarisation involved the first author repeatedly reading the included articles and reviewing extracted data; this was aided by the prior screening and data extraction processes. Using the genomic data lifecycle proposed by GA4GH [7] as a thematic framework, differences impacting genomic data governance and management in clinical and research settings were identified from the articles. These were then indexed and synthesised to describe the genomic data governance and management challenges across clinical and research settings.

### 2.4 Consumer Advisory Group Contributions

Preliminary findings on differences challenging genomic data across research and clinical care were presented at a LINEAGE‐GUARD Community Advisory Group meeting. While the identified genomic data challenges were substantiated by the LINEAGE‐GUARD members’ lived experience, their reflections and questions helped elucidate identified areas of difference. One example was the different understandings and views on informed consent. This critical input further refined the interpretation of the extracted data, informing the development of the presented findings.

## 3 Results

### 3.1 Included Articles

Figure 1 presents the screening process. The database searches yielded 9528 articles. After 5688 duplicates were removed, title and abstract screening of 3840 articles excluded 3691 articles. The full texts of the remaining 149 articles were screened. After excluding 106 articles, 43 articles met the inclusion criteria.

**Figure 1:**
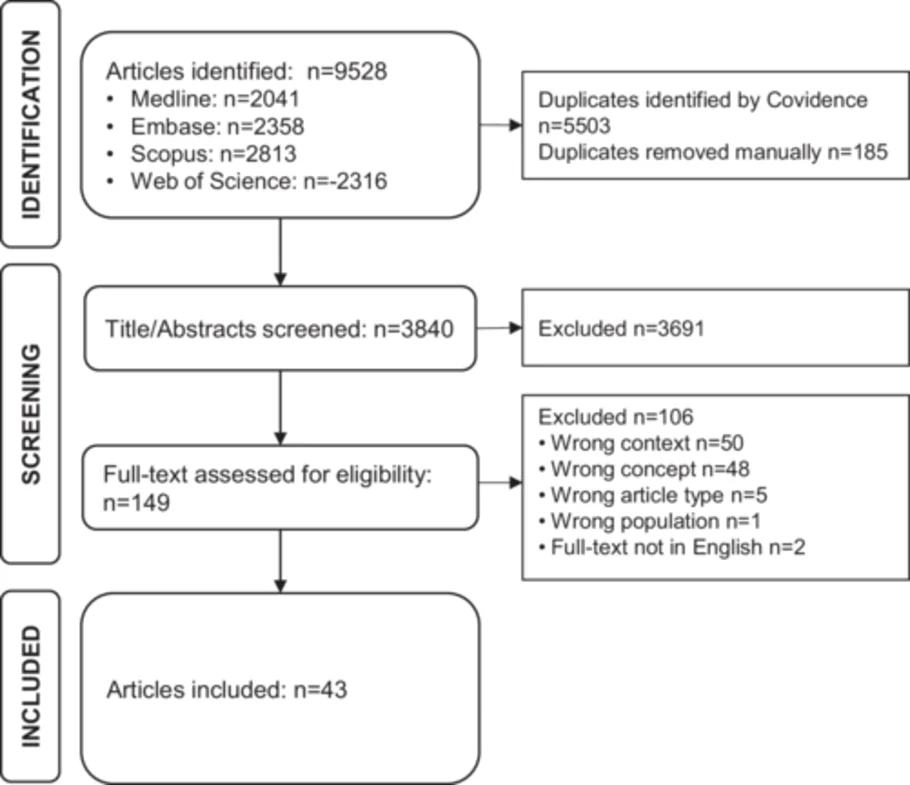
PRISMA flow diagram of article screening process.

#### 3.1.1 Characteristics of the Included Articles

Forty‐three articles were included for data extraction, analysis and synthesis to describe the differences impacting genomic data governance and management in clinical and research settings. Three types of evidence were included: empirical research (*n* = 15), legal research (*n* = 3) and theoretical/commentary articles (*n* = 25). Each evidence type was analysed separately to ensure rigour. To enhance the generalisability of the findings and address the research question, we focused on areas of difference that were recognised to affect genomic data governance and management across clinical and research settings. One of the broad themes that we identified in the articles was the varying perspective of genomics implementation in research and clinical practice, depending on positionality (e.g. whether through the voice of patient, carer, clinician, researcher, policy maker, etc). Additionally, the type of article (e.g. the format of the evidence source) dictated the way evidence was used and presented. We therefore analysed articles according to type of articles as well as paying attention to whose perspective was being presented. We will first present the findings from empirical research, legal research and then the theoretical/commentary articles.

### 3.2 Empirical Research Articles: Community Expectations vs Workforce Practices

Fifteen empirical research articles reported findings on lived experiences and views regarding genomic data in clinical and research settings (Table 2). Several research methods were used among these articles: qualitative methods (*n* = 6), mixed methods (*n* = 6) and quantitative methods (*n* = 3). Two perspectives were represented: the ‘community’ and the ‘workforce’. The ‘community’ perspective represented people who might provide their genomic data for clinical and/or research purposes: patients, research participants, family members/carers and the public. The ‘workforce’ perspective represented people who engaged with genomic data in their professional role: clinicians, researchers and other professionals. Eight articles reported findings from the ‘community’ perspective, six from the ‘workforce’ perspective, and one article reported findings from both perspectives.

**Table 2:** Overview of included empirical research articles (*n* = 15).

Reference	Jurisdiction	Publication year	Approach; Methods	Perspective represented	Data source (description)
[20]	Hong Kong	2023	Quantitative; survey	Workforce & Community	Healthcare professionals, parents of patients
[21]	Netherlands	2022	Mixed methods; literature review, questionnaire, interviews	Workforce	Next generation sequencing (NGS) stakeholders
[22]	United States of America	2015	Qualitative; interviews	Workforce	Genetic counsellors and research coordinators
[5]	United States of America	2018	Qualitative; interviews	Community	Participants [recruited from another research study]
[4]	United Kingdom	2018	Qualitative; interviews	Workforce	100,000 Genomes Project stakeholders ([Genomics England staff, associate members, and relevant stakeholder representatives]
[23]	Norway	2022	Quantitative; survey	Community	Patients [recently discharged from hospital]
[24]	Canada	2016	Qualitative; interviews	Workforce	Stakeholders in a clinical genomics research project
[25]	United States of America	2014	Mixed Methods; document analysis	Workforce	Consent forms used in Clinical Sequencing Exploratory Research (CSER) & Return of Results (RoR) studies [used in clinical research genomics studies]
[26]	Germany	2023	Quantitative; survey	Workforce	Physicians [who returned research results to participants/patients in clinical practice]
[27]	Hong Kong	2022	Mixed Methods; survey, focus groups	Community	Parents (Hong Kong Chinese parents of children 0–3 years or expecting parents)
[28]	Singapore	2020	Qualitative; focus groups	Community	General public
[29]	Australia	2024	Mixed Methods; document analysis & survey	Community	Consent forms, patients [received research‐funded genomic testing within clinical care]
[30]	Multiple	2016	Qualitative; focus groups	Community	Patients [Rare disease (RD) from RD‐Connect across 16 countries]
[31]	United States of America	2019	Mixed methods; survey	Community	General public
[32]	Australia	2020	Mixed methods; survey	Community	General public

Analysis identified reported differences in how the ‘community’ and the ‘workforce’ considered two genomic data governance and management topics: consent and the return of benefit, particularly for research participants. These differences are summarised in Figure 2 and discussed below.

**Figure 2:**
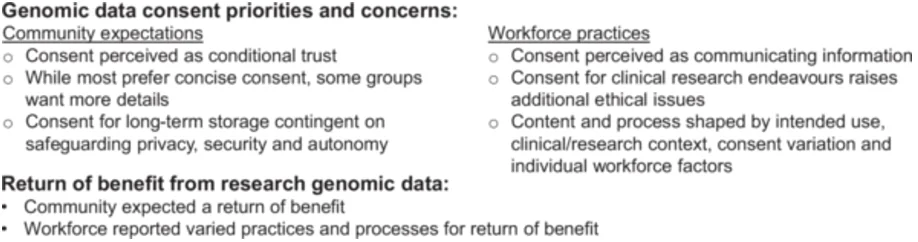
Comparison of community expectations versus workforce practices on key challenges impacting genomic data governance and management.

#### 3.2.1 Genomic Data Consent Considerations and Priorities

The community and the workforce were reported to have different perceptions and priorities regarding genomic data consent. These differences shaped their respective expectations of how genomic data should be governed and managed across clinical and research settings. Articles reporting community views primarily focused on privacy concerns and what happens to genomic data beyond its initial purpose. Comparatively, the workforce's experiences and perspectives focused on the process and context of obtaining informed consent.

##### 3.2.1.1 Community Expectations

Findings reported that the community viewed consent as conditional trust for the use and management of their genomic data. This reflected their expectation of what should happen to genomic data once consent is provided. While these expectations were similar across clinical and research settings, altruism was a distinguishing factor of research consent [5, 27] as a direct benefit was not guaranteed from research participation.

Most participants [5, 29], members of the public [31], and some patient cohorts [23] generally preferred a concise consent approach ‐ such as an abbreviated consent form [31] ‐ and were open to broad consent for future research uses of genomic data. However, this openness was conditional and context‐specific, particularly when data collected in clinical care were later used for research, or when research data were used for purposes beyond the original consent scope. For example, rare disease patients wanted specific details on data governance and protections before giving consent and wanted to be re‐contacted if their data were to be used for purposes beyond the original consent [30]. Similarly, 90% of surveyed Hong Kong parents wanted to be informed and asked for consent for additional uses of their child's biospecimen with 74% preferring an opt‐in consent approach [27].

Studies reported that patients and their families [20, 23, 27, 29], participants [5] and the public [28, 31, 32] were receptive to (long‐term) storage of clinical and research genomic data and for it to be shared and used for secondary (research) purposes. However, they raised concerns in providing such consent, wanting assurance that their privacy would be ensured, data secured and autonomy respected [20, 28, 30, 31]. Respect for autonomy meant that genomic data would be used as outlined by the obtained informed consent. For example, rare disease patients felt that any access and/or usage of their genomic data not outlined during informed consent threatened their autonomy [30]. These concerns were also raised about sharing genomic data in medical records for research purposes [32]. Thus, consent represented the community's trust that their privacy and autonomy would be protected in exchange for providing genomic data for clinical and/or research purposes.

##### 3.2.1.2 Workforce Experiences and Practices

The workforce viewed informed consent as an integral communication process aimed to ensure patients and participants were sufficiently informed of the potential benefits and risks in providing genomic data for clinical or research purposes. This was reflected in studies focusing on the workforce's practice and process of obtaining informed consent for clinical care or research studies.

While there were similarities across clinical and research consent practices and processes, additional considerations were identified when obtaining informed consent for combined or ‘*hybrid*’ clinical‐research purposes; these endeavours were recognised to create ethical tensions [4, 22, 24]. Notable practice considerations included explicitly framing the scope of consent and potential risks and benefits [4, 22, 24] and determining an appropriate threshold of understanding for consent to be valid [4, 22].

The five studies reporting the workforce's experiences of clinical and research genomic data consent focused on factors shaping the practice and process of obtaining informed consent [4, 21, 22, 24, 25]. Factors affecting the process of obtaining informed consent included the intended use of genomic data [4, 21, 24], whether consent was obtained as part of a clinical service or a research protocol [22] and the experience and practice of the workforce [22]. One study in the United States revealed how consent forms may influence workforce practices, attributing the considerable variation observed among nine consent forms to their respective study's design, scope and aim [25]. Furthermore, the authors suggested that consent variation could affect the community's expectations of how clinical and research genomic data are managed [25], including data storage and return of results. Hence, the workforce viewed consent as an integral part of their genomic data practice. Framed by the clinical and/or research consent context, this conversation aimed to ensure that patients and potential participants understood the implications of providing their genomic data for the intended activity.

Thus, the community's perception of consent representing their conditional trust for providing genomic data differed from the workforce's more functional‐orientated perception. While the community were focused on what happens to genomic data after consent is given, the workforce focused on how valid consent is obtained in different contexts. This observation suggests misaligned priorities and concerns about consent between the two groups, affecting their respective understanding and beliefs on how genomic data should be governed and managed in research and clinical care.

##### 3.2.1.3 Expectations on Returning Results From Genomic Research

Community and workforce views also differed about the return of results from genomic research data. These articles suggested that the community expected a return of results from their genomic data for research. The workforce, however, recognised the (until recently) longstanding convention of not guaranteeing a return of results from research participation. There was a notable shift in favour of returning some results arising from genomic research data, as reflected by its consideration among the workforce and in research protocols [4, 24, 25]. Both groups seemed to have similar views on returning results from clinical genomic data; a return of benefit was expected from clinical genomic data as clinical care aims to directly benefit patients.

##### 3.2.1.4 Community Expectations

The community reported an expectation of receiving a result when providing genomic data for research. Expectations ranged from receiving results from genomic research data [5] to receiving clinically relevant and/or actionable information [20]. Although most community representatives in these studies cited altruism as a motivator for agreeing to provide genomic data for additional research purposes, some also hoped or expected some benefit [5, 22, 23, 27].

Several studies acknowledged that this expectation may be due to the context of genomics research recruitment, namely participants being recruited for research studies in clinical settings and/or by their treating clinician [4, 5, 22, 24]. Interestingly, Berrios and colleagues [5] reported the unintentional yet important influence of pre‐existing clinical experiences on participants’ trust in research(ers) and expectations of participating in research studies. They found that participants with a pre‐existing clinical relationship with the enroling researcher and/or institute often extended their trust to the rest of the research team [5]. Furthermore, most participants attributed their expectations of receiving research results on their previous experiences of receiving *clinical* genetic testing results [5], despite the different context and the lack of explicit description of how or whether results would be returned.

#### 3.2.2 Workforce Experiences and Practices

Workforce perspectives on the return of results from genomic research data related to the primary aims and professional obligations associated with clinical and research activities. Several studies emphasised that researchers are not obliged to return any (genomic) results to participants [4, 5, 22, 24, 25]. How the workforce approached returning research genomic results to participants varied. Reasons for not returning research genomic results included not being included in the research design or protocol and the consent form [25]. In one study that did include returning genomic research results in its research design, 89 physicians (65.9%) reported that they did not return results to the patient; the most prevalent reason cited being the results’ lack of relevance to the patient or their family (38.2%) [26].

The boundaries shaping these conventional obligations were observed to become blurred for workforce involved with translational genomics studies. These studies reported mixed practices on returning results and differing perspectives on whether they should be returned [4, 24, 26]. For example, Frizzo‐Barker and colleagues reported that among their study's participants, ‘*many decisions about the return of results are currently made on an ad hoc basis*’ [24]. The lack of consensus regarding the return of research results was reported to add to the ethical tensions experienced by this workforce.

Thus, the community's expectations of receiving benefit from research genomic data differed from the workforce's perceived lack of obligation to return results to participants.

### 3.3 Legal Research Articles: Regulatory Regimes Shape Genomic Data Practices and Access Rights

Table 3 provides an overview of the legal research articles. These articles provide broader insights contributing to the differences reported among the empirical research articles by examining how legal regimes respectively shaped research and clinical genomic data governance and management practices.

**Table 3:** Overview of included legal research articles (*n* = 3).

Reference	Jurisdiction	Publication year	Examined regulations	Analysis focus	Conclusion
[33]	Ontario, Canada	2021	Personal Health Information Protection Act, 2004, SO 2004, c 3, Sch A (‘health privacy law’), complemented by Ontario Regulation 329/04. Freedom of Information and Protection of Privacy Act, RSO 1990, c F.31 (‘FOI law’)	Parents’ legal rights to access their child's raw genomic data in clinical and research contexts	Based on Canadian law, the authors conclude that parents generally have a stronger legal right to access their child's genomic data under the ‘health privacy law’ than the ‘FOI law’. Overall, parents’ legal right to access their child's health genomic data is stronger compared to child's research genomic data.
[34]	United States of America	2015	Mature minor legal notion under the US Constitution Health Information Technology for Economic and Clinical Health (HITECH Act) Health Insurance Portability and Accountability Act (HIPAA) Parents’ Bills of Rights (Arizona and Oklahoma)	Minors’ legal rights and protections of their decisions involving genomic data	The author concludes that the law provides little protection for minors to make decisions regarding genomic testing and who can access the results. However, the mature minor exception can be a safeguard for unemancipated minors. Minors have a bit more control in research settings as they need to consent to participate whereas parental governance is usually accepted in clinical settings.
[35]	United States of America	2018	Health Insurance Portability and Accountability Act of 1996 (HIPAA) Privacy Rule, amended in 2013	HIPAA privacy tiers and its impact of the minimum necessary standard on genomic data sharing across different domains	While HIPAA's four tiers of privacy protection shape the use of (genomic) health data and the data subject's control on who can access their data, the authors determined that there are no clear guidance on how to apply them.

These articles highlighted how legal and regulatory regimes governing research and clinical care respectively shape genomic data practices and access rights. Although these articles are drawn from the United States [34, 35] and Canada [33], they illustrate a broader point: clinical and research contexts may be associated with different legal rules regarding access to, use of, and decision‐making authority over genomic data. For example, Evans and Jarvik's legal analysis of the United States’ 2013 revision of the Health Insurance Portability and Accountability Act of 1996 (HIPAA) Privacy Rule's minimum necessary standard concluded that the lack of clear regulatory guidance could challenge how genomic data, particularly clinical data, are accessed and shared in practice [35]. Also based on the laws of the United States, Clayton examined how the two legal regimes respectively protected (or not) minors’ genomic data choices, concluding that there was ‘*remarkably little protection for minors who wish to make decisions about genetic and genomic testing and to control who has access to the results*’ [34]. Similarly, Beauvais and colleagues used three case scenarios to illustrate how the laws of Ontario, Canada and the purpose of the collected genomic data determined parents’ legal rights to access their child's raw genomic sequence data [33]. Together, the legal articles complement the empirical findings by showing that differences between research and clinical genomic data practices are also structured by law and regulation.

### 3.4 Theoretical/Commentary Articles: Addressing Challenges Impacting Clinical and Research Genomic Data Governance and Management Practices

Twenty‐five theoretical/commentary articles focused on issues and/or challenges impacting genomic data governance and management practices across clinical and research settings (Table 4; refer to Supplementary material 3 for the comprehensive summary). All were authored by the genomic workforce; notably, clinicians, researchers, scientists and other individuals who engaged with genomics for their professional practice. Articles predominantly represented experiences and perspectives from the United States (*n* = 14) and other high‐income jurisdictions, including Canada (*n* = 3), the European Union (*n* = 2), and the United Kingdom (*n* = 2). Two commentaries were authored by members of GA4GH. The term ‘commentators’ is used to reflect the perspectives reported in these articles. Based on the articles’ corresponding author and authorship affiliations, none were identified to be primarily authored by community representatives.

**Table 4:** Overview of included theoretical/commentary articles (*n* = 25).

Reference	Jurisdiction	Publication year	Focus
[36]	United States of America	2018	Proposes clinical genomic data elements to be shared by clinical laboratories
[37]	Canada	2020	Describes rare disease data stewardship (from consent to disposal)
[38]	United States of America	2013	Proposes the argument for pursuing incidental findings in clinical and research genomic data
[39]	United States of America	2015	Describes data sharing among the Undiagnosed Disease network (UDN)
[6]	European Union & United States of America	2015	The risks and benefits considering the sensitive and digital nature of genomic information
[40]	United States of America	2017	Proposes principles for building a shared medical info commons
[41]	United States of America	2017	Explores the case for research participants accessing and sharing raw research genomic data
[42]	North America	2020	Reviews the importance of (genomic) data reanalysis and sharing for diagnosis among Undiagnosed Rare Disease patients
[2]	United Kingdom	2019	Argues for research and clinical practice to co‐exist
[43]	United States of America	2014	Proposes principes for returning research results ‐ minimum to maximum levels of information ‐ to participants
[44]	United States of America	2017	Proposes standards to govern research use of clinical genomic data stored in electronic medical records (EMR)
[45]	N/A	2014	Proposes a human rights approach to govern international genomic data sharing
[46]	United States of America	2017	Explores the genomic data policy and practice implications of the 21st Century Cures Act (‘the Act’),
[47]	United States of America	2018	Highlights the ELSI policy issues associated with genomic data in implementing a learning health system
[48]	GA4GH	2018	Proposes principles for responsible paediatric genomic data sharing
[7]	GA4GH	2021	Describes GA4GH and their work to facilitate genomic data sharing via standards and frameworks
[49]	United States of America	2018	Describes the privacy and confidentiality risks associated with genomic databases
[50]	Belgium	2021	Explores the ELSI implications of (genomic) data collected using non‐traditional methods
[51]	N/A	2023	Explores the ethical issues associated with genomic data and testing across research, clinical and individual use
[52]	European Union	2020	Considers the ethical and legal challenges associated with genomics, describes ‘best practices’ addressing these issues often through legislation
[53]	Canada; European Union	2022	Examines the ethical and legal debate in precision cancer medicine and translational sciences
[54]	United States of America	2015	Proposes a translational approach to ethics for genomics
[55]	United States of America	2020	Proposes principles to address legal conflicts between competing legal frameworks
[56]	Netherlands	2021	Identifies a combined research‐care approach to address the core needs of a learning health system
[57]	United Kingdom	2019	Proposes ‘best practices’ for addressing the potential identification of misattributed parentage

Analysis reiterated how differences between clinical and research settings challenged the workforce's genomic data governance and management practices, and how they amplified the ethical, legal and social issues (ELSI). The articles focused on describing genomic data governance and management practices of rare disease and undiagnosed patient cohorts (*n* = 3) and examining ELSI arising from genomic data practices across clinical and/or research settings (*n* = 22). Among the latter, commentators examined ELSI considerations for specific genomic data lifecycle stages, consent, return of results from genomic data and advancing precision medicine. Drawing from their authorship's experience and expertise, fourteen articles also presented potential ‘solutions’ to address genomic data governance and management challenges.

### 3.5 Genomic Data Governance and Management Among Rare and Undiagnosed Disease Cohorts

Three theoretical/commentary articles described the genomic data governance and management practices of rare and/or undiagnosed disease patient cohorts in North America [37, 39, 42]. All acknowledged the unique relationship between genomics and these cohorts; particularly the importance of sharing clinical and research data in facilitating a diagnosis and informing clinical care. Each article described how its respective cohort benefited from integrating research opportunities and genomic technologies to address the limitations of clinical workflows [37, 39, 42]. However, as noted by Bernier, there is ‘*significant overlap across clinical care, research, and quality assurance activities*’ in the rare disease healthcare context, challenging longstanding assumptions underlying data privacy laws and research ethics [37]. To address these challenges, commentators emphasised the importance of establishing robust infrastructure and transparent governance in enabling ethically appropriate and legally responsible genomic data sharing between clinical and research settings and/or organisations. For example, Brownstein and colleagues described how and why the Undiagnosed Diseases Network developed its own Data Sharing and Use Agreement; this agreement outlined the data governance and management conditions governing all activities [39]. Together, these articles highlighted how rare and undiagnosed disease patient cohorts may particularly benefit from clinical and research genomic data, leveraging the generated information to advance scientific understanding and improve patient care.

### 3.6 Workforce Genomic Data Practices Are Shaped by Ethical, Legal and Social Implications

Twenty‐two theoretical/commentary articles examined how ELSI associated with genomic data shaped workforce practices across clinical and research settings. Commentators discussed how privacy risks, ethical concerns and legal ramifications, within and across clinical and research settings, affected workforce genomic data management practices. Thus, these commentators considered the policy and practice implications of these issues and how they would or could affect genomic data governance and management.

Commentators discussed how consent considerations shaped the workforce's clinical and research practice across the genomic data lifecycle. The overarching regulatory differences respectively governing clinical and research settings were recognised to determine the purpose of obtaining informed consent [2, 36, 37, 41, 42, 43, 47, 48, 49, 51, 53, 54, 55, 57]. Rahimzadeh and colleagues stated that ‘*in strictly legal terms*’, informed consent is provided for a specific purpose; to release information for clinical care and to participate in research studies [48]. Another noted difference related to the comparatively narrow scope of clinical consent for using genomic data beyond its initial purpose [53, 55]. Winkler and Knoppers also noted that the scope of research consent was often broad, accounting for the potential future use, analysis and sharing of research genomic data [53]. Additionally, commentators examined how consent enabled and challenged how the workforce could collect [50], store [49], access [6], use [51], share [6, 36, 50] and disclose [57] clinical and research genomic data respectively.

Despite the different clinical and research governance regimes, similar consent considerations were attributed to shaping the workforce's genomic data practices, namely safeguarding data privacy, confidentiality and security [6, 36, 49, 50, 57]. In addition to adhering to clinical and research governance regimes, these considerations also reflected the sensitive, identifiable and digital nature of genomic data and the need to respect patient/participant autonomy. Thus, differences in the scope of clinical and research consent were attributed to creating genomic data practice challenges for workforces working across both settings.

#### 3.6.1 Proposed ‘Solutions’ to Address Genomic Data Governance and Management Challenges and Conflicts

Fourteen commentaries included suggestions to address challenges affecting genomic data governance and management practices. Primarily based on the commentators’ experience and expertise, these proposed ‘solutions’ aimed to address current and foreseen practice gaps and ethical challenges experienced by the workforce. Proposed ‘solutions’ related to three challenges: considering how consent can complicate the reality of genomic data practice; deciding to return results derived from research genomic data; and navigating and addressing the clinical‐research dichotomy to advance precision medicine (Table 5). Most proposed ‘solutions’ aimed to maximise the clinical and scientific potential afforded from clinical and research genomic data. For example, proposed ‘solutions’ aimed to advance scientific and clinical understanding by enabling and facilitating responsible clinical and/or research genomic data use [38, 44] and sharing [7, 36, 45, 48].

**Table 5:** Challenges impacting clinical and research genomic data governance and management and proposed ‘solutions’, as identified in theoretical/commentary articles.

Genomic data challenge	Proposed ‘solutions’
Considering how consent can complicate the reality of genomic data practice	The original consent process should specify and address how genomic data will be managed and discuss any privacy and confidentiality risks and considerations, e.g. The possibility of identifying misattributed parentage Genomic data sharing should protect the identity of the individual
Deciding to return results derived from research genomic data	The possibility and option of receiving (or not) clinically actionable and verified genomic results should be discussed during the research consent process. Research protocols should account for the possibility of returning genomic results and any follow‐up actions
Navigating and addressing the clinical‐research dichotomy to advance precision medicine	A new ethical framework and legal approach for translational genomics that acknowledges the interdependent relationship between research and clinical care should be established. The ‘social contract’ dichotomy of clinical and research endeavours should be re‐negotiated to acknowledge the translational nature and activity of genomics

#### 3.6.2 Considering How Consent Can Complicate the Reality of Genomic Data Practice

Genomic data practices became complicated when differences were identified between the consent scope and the reality of professional practice in research and clinical care. The practical genomic data applications, constraints and implications these commentaries discussed focused on situations in which workforce members worked across clinical and research settings, and when genomic data could be used for purposes not addressed in the original consent process. One example related to a policy gap in the United States regarding sharing clinically obtained genomic data. Recognising that clinical laboratories cannot contact patients to obtain consent for sharing clinical data with public genomic databases, Azzariti and colleagues proposed specific genomic data governance elements that encouraged data sharing whilst protecting the identity of the individual patient [36]. Other consent practice ‘solutions’ related to explicitly discussing the inherent privacy and confidentiality risks of genomic data with the patient/participant and how these risks would be proactively managed and mitigated in practice [6, 49, 50], and the possibility of identifying misattributed parentage from research genomic testing [57]. These consent and ethical considerations highlighted the practice challenges that needed to be navigated by the clinical and/or research workforce.

#### 3.6.3 Deciding to Return Results From Research Genomic Data

Two commentaries focused on the return of results to research participants and its implications on workforce practices [41, 43]. Drawing from their collective research programme experiences in the United States, Jarvik and colleagues proposed practice and policy recommendations for returning (or not) research genomic results to participants; outlining the circumstances and rationales for returning minimal information (‘*the floor*’), all information (‘*the ceiling*’), and any level of information in between [42]. Similarly, Haga and colleagues highlighted the workforce practice implications in considering the possibility of research participants accessing their raw genomic data [41]. Although both articles noted the practical limitations reinforcing researchers’ lack of obligation for returning results or data to research participants, they acknowledged the emerging conversation of returning benefit, including results, from research genomic data to research participants.

#### 3.6.4 Navigating and Addressing the Clinical‐Research Dichotomy to Advance Precision Medicine

Six commentaries explored the ethical and legal ramifications of clinical and research genomic data practices in advancing precision medicine. Commentators noted how genomic data spanning clinical and research endeavours – or ‘*translational sciences*’ [54, 55] – can blur the boundary between these settings. To help navigate the conflicting clinical and research regimes and frameworks encountered in practice, commentators proposed ‘solutions’ to address the ethical, legal and social challenges associated with these settings.

Three articles proposed guidelines and principles aimed at addressing the ethical tensions and conflicting legal frameworks between clinical and research settings [54, 55, 56]. All discussed the limitations of the current clinical and research governance regimes in managing genomic data, facilitating translational sciences and advancing precision medicine [54, 55, 56]. Notably, Wolf and colleagues argued that genomics and translational sciences warranted new ethical and legal approaches which addressed and resolved governance conflicts whilst protecting the rights of the individual [54, 55]. These were central in their proposals for developing an ethical framework [54] and legal principles [55] for translational sciences; both proposals aimed to resolve conflicts caused by the different ethical obligations and legal frameworks respectively governing clinical and research settings.

Also acknowledging genomics, translational sciences and precision medicine, the remaining three articles suggested a re‐negotiation of the ‘social contract’ [2, 47, 53]. The social contract is a symbolic agreement between the community and an authority figure that outlines the overarching principles and conditions for a particular activity. The social contract is often conveyed as informed consent in clinical and research endeavours, outlining the agreed actions and expectations between the community and clinicians/researchers. The articles recognised how genomics, as a translational science, alluded to a symbiotic relationship between research and clinical care [2, 47, 53]. Horton and Lucassen argued that this feature challenged the conventional social contract between clinical care and research; instead, they suggested the two practices are better represented along a spectrum [2]. They also surmised that re‐thinking or re‐negotiating the clinical and research ‘social contract’ could address or potentially resolve the identified ethical and legal tensions associated with genomic data and more broadly, facilitate the implementation of learning healthcare systems and precision medicine.

In summary, the theoretical/commentary articles highlighted a range of normative challenges shaping how the workforce engages with genomic data as part of their professional practice. While some challenges were attributed to differences between the oversight of research and clinical care, most were focused on how the ethical, legal and social implications challenged workforce genomic data practices. The ‘solutions’ proposed – in addition to the reported different expectations between the ‘community’ and ‘workforce’ ‐ indicate the importance of involving the community in genomics implementation initiatives to ensure all genomic data actors understand the different implications of providing consent for genomic data use in research and clinical care.

## 4 Discussion

This review provides insight into key challenges affecting genomic data governance and management practices across research and clinical care. By synthesising empirical, legal and theoretical evidence across these settings, this review highlights how differences in expectations, practices and policies can challenge genomic data governance and management practices and perceptions. Despite the legal [33, 34, 35] and some theoretical/commentary articles [7, 50, 52, 55] delineating the differences between research and clinical care regimes, it was acknowledged that because these differences were not always clear to those involved and/or affected, genomic data practices could nonetheless be challenged.

### 4.1 Misunderstandings and Misaligned Expectations Can Challenge Genomic Data Governance and Management Practices and Perceptions

The main challenge from the empirical articles was the observed misalignment between community and workforce expectations of genomic data, particularly where genomic research data are collected in a clinical context. Notably, there was a difference between what the community expected should be done with genomic data and how the workforce was reported to engage with genomic information in professional practice. This was reflected in each group having distinct priorities concerning informed consent and the return of data or results from genomic research data. While the community perceived consent as a conditional agreement, in which they entrusted their genomic data to be sufficiently protected and appropriately used for research or clinical care [5, 20, 27, 28, 29, 30, 31, 32], the workforce was reported to view consent as an important process of communicating the potential benefits and risks of providing genomic data [4, 21, 22, 24, 25]. Similarly, while the community were reported to not comprehend that, unlike providing genomic data for clinical purposes, genomics research participation does not oblige a return of results [5, 22, 23, 27], the workforce was cognizant of this difference [4, 5, 22, 24]. These findings suggest that the community were less informed of the different implications associated with clinical and research endeavours. It also highlights how differences in understanding can create unmet expectations, which can challenge how governance and management practices are perceived. Although the aim of any individual research study may have shaped the identified differences, these findings could also reflect each group's general genomic data priorities and concerns. For instance, previous research with patients, participants and the public echo similar concerns and expectations on how genomic data should be managed [58, 59]. Even though these findings highlighted different consent priorities and expectations of returning results from genomic research data between the community and the workforce, this misalignment alludes to other possible differences in expectations and/or understanding within the genomics community.

Compared with the workforce, these findings indicate that the community were less informed of the genomic data implications respectively associated with clinical and research endeavours. This was illustrated by four studies reporting their participants having some expectation of receiving a return of results from genomics research [5, 22, 23, 27]. This misunderstanding could be attributed to therapeutic misconception, when individuals falsely assume that the primary aim of clinical research is to inform their personal health care [60]. As such, therapeutic misconception has been suggested to potentially bias clinical genomics research participation [61] which could undermine the voluntary intent of informed consent for research participation [62]. Informed consent intends to mitigate this by ensuring individuals understand and appreciate what they are providing their consent for whilst supplying the workforce with the means to address any identified differences in understanding and/or expectations that may subvert an individual's decision‐making. Thus, these findings reinforce the importance of transparent informed consent in research and clinical care, ensuring the parameters shaping genomic data governance and management are appropriately communicated and understood.

### 4.2 Amplifying the Community Voice in Genomic Data Governance and Management

The theoretical/commentary articles analysed in this review provided insights on the perspectives represented in the published literature. These articles primarily focused on ELSI‐related genomic data practices across clinical and research settings. It is important to note the authorship of these commentaries. All were authored by those with a professional role in or around genomics, describing their experiences and perspectives engaging with genomic data. While the community's interests and priorities were discussed, none of these articles were authored by community representatives. Furthermore, none of the proposed ‘solutions’ were from the community. Identifying this gap is important as it may provide insights into the different genomic data expectations observed between the community and the workforce. Although this authorship gap may reflect academic publishing norms, it also highlights the inherent power imbalance between the community and the workforce across research and clinical care. Addressing this gap is particularly important in protecting community interests, and professional trust and confidence in governance. One solution could be to ensure community input is sought in developing genomic data governance.

The importance of community involvement in realising the benefits of genomic medicine and research is recognised globally [63, 64, 65]. The genomic data challenges – such as the observed misaligned expectations ‐ and workforce proposed ‘solutions’ identified in this review indicate that more work is needed to ensure community insights are considered. As a key actor of genomic data, involving the community in developing genomic data governance and policies is warranted to ensure their interests are protected and concerns are addressed. This involvement could not only identify and address any potential misunderstandings of how genomic data are governed and managed but also enhance community trust and confidence; critical for developing robust genomic data governance and policies that are fit‐for‐purpose.

Meaningful community involvement is essential in creating more equitable healthcare systems. This is particularly important in genetics/genomics, an area historically associated with racism, discrimination and other structural barriers [66]. Several guidelines have been developed, providing advice to establish trust and build relationships to meaningfully involve the community in genomics research and implementation activities [66, 67, 68, 69]. In addition to ensuring the community perspective is integrated to inform these efforts, meaningful involvement could help build community trust in genetics/genomics, particularly among historically underrepresented and underserved populations. Recognising its importance, community/consumer involvement with genomics health research and implementation programmes are increasingly being encouraged and, in some instances, mandated by health research funders and organisations [63]. Meaningful partnerships empower communities to share their lived experiences, priorities and concerns to shape the work, objectives, and outcomes of genomics research and implementation initiatives. For example, LINEAGE ‐ in developing an empirically informed and normatively robust genomic data governance framework for Australia ‐ has partnered with a community advisory group, the GUARD, to embed lived experience expertise and insights across its research programme and foster authentic partnerships across the Australian genomics ecosystem. The lived experience expertise of the GUARD is embedded in this review, highlighting the importance and impact of meaningful partnerships in creating robust genomic data governance.

### 4.3 Strengths and Limitations

This review identified differences that challenge genomic data governance and management across clinical and research settings as reported in the peer‐reviewed literature. It is important to acknowledge that relevant articles may not have been included for various reasons, including pragmatic decisions to not search the reference lists of the included articles and for one author to conduct title/abstract screening. We also acknowledge our findings, which are based on literature published since 2013, may not reflect current practice in all settings. This may be due to several reasons, including inconsistent terminology, discrepancies between law and practice, international variation, or recent changes/advances. With nineteen of the 43 included articles from the United States, some findings may not be applicable or transferable to other jurisdictions. Despite these limitations, this review identified important gaps and considerations for developing robust genomic data governance. Guided by the pragmatic paradigm, we synthesised empirical and non‐empirical literature to provide actionable considerations to inform the development of a genomic data governance framework.

## 5 Conclusion

This review identified how differences in understanding and expectations between genomic data actors, as well as conflicting perceptions of responsibilities and entitlements in research and clinical care regimes, can challenge genomic data governance and management practices. Community expectation of how genomic data should be managed in research and clinical care differed from workforce genomic data practices. Thus, the community may assume the implications of providing genomic data in research and clinical care are similar, when they are not. Although the theoretical/commentary articles examined the ethical, legal and social implications associated with genomic data practices across research and clinical care, none were authored by the community. Meaningful community involvement could enhance trust in genomic medicine and research, identify differences in understanding and expectations. Addressing these challenges in transparent guidance documents and governance frameworks is important in ensuring community trust in genomic medicine and precision medicine. Given the global recognition of meaningful community involvement in genomics health research and implementation, this review reinforces why partnering with communities will be critical in developing robust genomic data governance to effectively and responsibly leverage genomics to maximise benefits, minimise harms and, more broadly, progress towards more equitable health care.

## Author Contributions

**Alice Kim:** conceptualisation, investigation, writing – original draft, methodology, visualisation, writing – review and editing, formal analysis, project administration, data curation. **Bronwen Merner:** conceptualisation, investigation, methodology, writing – review and editing, formal analysis, project administration, supervision, data curation. **Mark Taylor:** conceptualisation, investigation, funding acquisition, methodology, writing – review and editing. **Yingyi Luo:** investigation, writing – review and editing. **Fabian Cannizzo:** investigation, writing – review and editing, methodology. **Alan Petersen:** investigation, writing – review and editing, funding acquisition, methodology. **Clara Gaff:** conceptualisation, investigation, funding acquisition, methodology, writing – review and editing. **Ainsley J. Newson:** conceptualisation, investigation, funding acquisition, writing – review and editing, methodology. **Kate Marchant:** investigation, writing – review and editing. **Louse Keogh:** conceptualisation, investigation, writing – review and editing, methodology, supervision, project administration, formal analysis, funding acquisition.

## Conflicts of Interest

The authors declare no conflicts of interest.

## Supporting information


Supporting File 1

Supporting File 2

Supporting File 3

## Data Availability

All data generated or analysed during this study are included in this published article [and its supplementary information files].
