# Correction to “Genomic Data Governance Challenges Between Research and Clinical Care: A Scoping Review”

**DOI:** 10.1111/hex.70934

**Published:** 2026-10-13

A. Kim, B. Merner, M. Taylor, et al., “Genomic Data Governance Challenges Between Research and Clinical Care: A Scoping Review,” *Health Expectations* 29 (2026): e70889. https://doi.org/10.1111/hex.70889.

The corresponding author's name was misspelled and has been corrected from “Louse Keogh” to “Louise Keogh.”

The online version of this article has been corrected accordingly.

We apologize for this error.

