# Supplementary material for: Genomic Data Governance Challenges Between Research and Clinical Care: A Scoping Review
Source: Health Expect. 2026 Oct 3;29(5):e70889. doi: 10.1111/hex.70889 (PMC13633702; doi:10.1111/hex.70889)
Supplement: Supplementary file 1 [file HEX-29-e70889-s001.docx]

Supplementary material 2. Article screening inclusion and exclusion criteria.

| **Criteria** | **Inclusion** | **Exclusion** |
| --- | --- | --- |
| 1. **Published date range** | 2013 (when the Global Alliance for Genomics and Health, GA4GH was formed) to present | Before 2013 |
| 2. **Publication language** | English language | Non-English language for abstract or full-text |
| 3. **Study type** | Database searches   - Empirical qualitative, quantitative, and mixed methods study (including description of data collection and analysis methods) - Opinion piece, perspective, commentary, protocols   Grey literature   - Reports, websites of governments and other relevant organisation | - Preprints - Editorials - Reviews, with explicit methods (eg. systematic reviews) - Dissertations/Thesis - Protocols (if no data) - Poster/conference abstracts - Correspondence - Books/Book chapters - Lecture notes |
| 4. **Participants** | Any stakeholder in **human genomic or genetic data**.  Includes but is not limited to:   - Clinicians – doctors, genetic counsellors, allied health - Laboratory scientists – variant curators, bioinformaticians, … - Researchers - Consumers and patients - Policymakers - The community and public | The participants discuss **non-human** genomic or genetic data. Eg. animal, bacteria, virus, … |
| 5. **Concept** | The article involves **any aspect of the management** of human genomic or genetic data**.**  Including but limited to genomic data:   - Consent - Collection - Storage - Custodianship / Stewardship - Access - Sharing, Disclosure of (research) incidental findings - Usage, Processing - Other data-related matters | The article and/or findings do not involve human genomic data management and/or aspects of genomic data management.   - Development or description of human genomic data platforms/infrastructure - Development or description of human genomic data applications/apps - Development or description of human genomic data workflows - Development or description of human genomic databases - Methodologies involving human genomic data , eg. polygenic risk scores (PRS) - Molecular/laboratory investigations using human genomic data - Modelling of/using human genomic data - Attitudes towards genomic testing only - Knowledge of genomics/genetics - Evaluation of research participation experience - Evaluation of genomic testing as an intervention - Economic studies on genomic testing - Clinical guidelines/recommendations on genetic information disclosure - Biospecimen use that doesn’t explicitly discuss human genomic/genetic data - Primarily focus on health/medical/clinical data |
| 6. **Context** | The article discusses any aspect of the management of human genomic data in **both settings in the abstract.**   - Clinical settings (eg., health-related) - Research settings, including biobanks | The article:   a. discusses human genomic data management in only one of clinical or research settings (not both); b. Clinical genomic data management; c. Research genomic data management, including biobanks; d. does not discuss the management of human genomic data in either research or clinical settings in the abstract; e. only discusses DTC genetic/genomic testing; f. only discusses genomic testing for immigration purposes; g. only discusses genomic data management in forensic settings; h. only discusses genomic data management in egg/sperm/embryo donor settings; i. only discusses genomic data in (life) insurance settings; j. h. only discusses genomic data in educational settings |

**INCLUSION/EXCUSION DECISION-MAKING PROCESS:**

- Studies that have ‘y’ for all criteria = INCLUDE.
- Studies that have ‘y’ plus ‘maybe’ for any criteria = INCLUDE. Then in ‘notes’ tab, specify the number of the unclear criterion or criteria with a question mark e.g., “5?”. We will then discuss these so do not worry about rating the phenomenon at this point.
- Studies that have ‘n’ for one or more criteria = EXCLUDE
  - Reason for exclusion: Use the exclusion reason that comes first on the list e.g., if excluded because “wrong study design” and “wrong phenomenon,” stated exclusion reason would be “wrong study design.”
  - If you are unsure whether an excluded study meets a particular criterion, exclude it based on the criterion that you are certain about e.g., if you are unsure whether the study has a wrong population but know the study is ‘wrong phenomenon’ then exclude on ‘wrong phenomenon.’
