# Supplementary material for: Genomic Data Governance Challenges Between Research and Clinical Care: A Scoping Review
Source: Health Expect. 2026 Oct 3;29(5):e70889. doi: 10.1111/hex.70889 (PMC13633702; doi:10.1111/hex.70889)
Supplement: Supplementary file 2 [file HEX-29-e70889-s002.docx]

Supplementary material 1. Database search strategies.

| **Ovid MEDLINE(R) ALL <1946 to October 04, 2024>** | | **Results** |
| --- | --- | --- |
| 1 | (genomic or genomics).mp. | 432344 |
| 2 | ((genetic or genetics or gene or genes) adj3 (clinical or test or tests or testing or sequenc* or data or information or predictive or diagnostic or diagnosis or medical)).mp. | 381167 |
| 3 | 1 or 2 | 762722 |
| 4 | (governance or govern or governing or share or sharing or store or storage or storing or collect or collecting or collection or access or regulation or regulate or regulating or consent or confidentiality or privacy or security or stewardship or steward or processing or process or custodianship or custody or "secondary use" or "re-use" or "reuse" or "re-analysis" or "reanalyze" or "reanalyse" or "re-analyze" or "re-analyse" or "recontact" or "re-contact" or usage or utilization or utilisation or responsible or responsibility or disclosure).ti,kf. | 1068302 |
| 5 | ((governance or govern or governing or share or sharing or store or storage or storing or collect or collecting or collection or access or consent or confidentiality or privacy or security or stewardship or steward or processing or process or custodianship or custody or "secondary use" or "re-use" or "reuse" or "re-analysis" or "reanalyze" or "reanalyse" or "re-analyze" or "re-analyse" or "recontact" or "re-contact" or usage or utilization or utilisation or responsible or responsibility or disclosure) adj6 (genomic or genomics or ((genetic or genetics or gene or genes) adj3 (clinical or test or tests or testing or sequenc* or data or information or predictive or diagnostic or diagnosis or medical)))).mp. | 15804 |
| 6 | 3 and 4 | 26603 |
| 7 | 5 or 6 | 38950 |
| 8 | (clinic or clinical or clinician* or patient or patients or medicine or hospital*).mp. and ((biobank* or bio-bank* or participant*).mp. or exp research/ or research personnel/ or research*.ti,kf,ab.) | 1852897 |
| 9 | 7 and 8 | 3389 |
| 10 | ((management or manage or managing or regulation or regulating or regulate or "use") adj3 (genomic* or genetic) adj3 (data or database* or information or registr* or computing or biobank* or bio-bank* or test* or sequenc*)).mp. | 3265 |
| 11 | 10 and 8 | 647 |
| 12 | genomics/ | 71991 |
| 13 | (genomic or genomics or data or information or biobank*).ti,kf. | 587488 |
| 14 | ((genetic or genetics or gene or genes) adj3 (clinical or test or tests or testing or sequenc* or data or information or predictive or diagnostic or diagnosis or medical)).ti,kf. | 50599 |
| 15 | databases, genetic/ or databases, factual/ or genetic testing/ or genetic predisposition to disease/ or privacy/ or genetic privacy/ or informed consent/ or confidentiality/ | 392389 |
| 16 | 12 or 13 or 14 or 15 | 1002950 |
| 17 | 9 or 11 | 3864 |
| 18 | 17 and 16 | 2740 |
| 19 | limit 18 to english language | 2683 |
| 20 | limit 19 to yr="2013 -Current" | 2041 |

| **Embase Classic+Embase <1947 to 2024 October 08>** | | **Results** |
| --- | --- | --- |
| 1 | (genomic or genomics).mp. | 622070 |
| 2 | ((genetic or genetics or gene or genes) adj3 (clinical or test or tests or testing or sequenc* or data or information or predictive or diagnostic or diagnosis or medical)).mp. | 634606 |
| 3 | 1 or 2 | 1144972 |
| 4 | (governance or govern or governing or share or sharing or collect or collecting or collection or store or storage or storing or access or regulation or regulate or regulating or consent or confidentiality or privacy or security or stewardship or steward or processing or process or custodianship or custody or "secondary use" or "re-use" or "reuse" or "re-analysis" or "reanalyze" or "reanalyse" or "re-analyze" or "re-analyse" or "recontact" or "re-contact" or usage or utilization or utilisation or responsible or responsibility or disclosure).ti,kf. | 1318054 |
| 5 | ((governance or govern or governing or share or sharing or collect or collecting or collection or store or storage or storing or access or consent or confidentiality or privacy or security or stewardship or steward or processing or process or custodianship or custody or "secondary use" or "re-use" or "reuse" or "re-analysis" or "reanalyze" or "reanalyse" or "re-analyze" or "re-analyse" or "recontact" or "re-contact" or usage or utilization or utilisation or responsible or responsibility or disclosure) adj6 (genomic or genomics or ((genetic or genetics or gene or genes) adj3 (clinical or test or tests or testing or sequenc* or data or information or predictive or diagnostic or diagnosis or medical)))).mp. | 21291 |
| 6 | 3 and 4 | 40972 |
| 7 | 5 or 6 | 57854 |
| 8 | ((clinic or clinical or clinician* or patient or patients or medicine or hospital*) and (biobank* or bio-bank* or research* or participant*)).mp. | 3282973 |
| 9 | 7 and 8 | 6328 |
| 10 | ((management or manage or managing or "use" or regulation or regulating or regulate) adj3 (genomic* or genetic) adj3 (data or database* or information or registr* or computing or biobank* or bio-bank* or test* or sequenc*)).mp. | 4280 |
| 11 | 10 and 8 | 1007 |
| 12 | genomics/ | 95239 |
| 13 | (genomic or genomics or data or information or biobank*).ti,kf. | 722449 |
| 14 | ((genetic or genetics or gene or genes) adj3 (clinical or test or tests or testing or sequenc* or data or information or predictive or diagnostic or diagnosis or medical)).ti,kf. | 68271 |
| 15 | databases, genetic/ or databases, factual/ or genetic testing/ or genetic predisposition to disease/ or privacy/ or genetic privacy/ or informed consent/ or confidentiality/ | 392775 |
| 16 | 12 or 13 or 14 or 15 | 1178729 |
| 17 | 9 or 11 | 7097 |
| 18 | 17 and 16 | 4586 |
| 19 | limit 18 to (english language and yr="2013 -Current") | 3513 |
| 20 | limit 19 to conference abstract status | 1155 |
| 21 | 19 not 20 | 2358 |

| **Web of Science Search Queries, conducted October 10, 2024** | | **Results** |
| --- | --- | --- |
| 1 | AK=(database* or genetic* or privacy or consent or confidentiality) | 478942 |
| 2 | TI=((genetic or genetics or gene or genes) NEAR/2 (clinical or test or tests or testing or sequenc* or data or information or predictive or diagnostic or diagnosis or medical)) OR AK=((genetic or genetics or gene or genes) NEAR/2 (clinical or test or tests or testing or sequenc* or data or information or predictive or diagnostic or diagnosis or medical)) | 70726 |
| 3 | TI=(genomic or genomics or data or information or biobank*) OR AK=(genomic or genomics or data or information or biobank*) | 2185501 |
| 4 | #1 OR #2 OR #3 | 2641238 |
| 5 | TS=((governance or govern or governing or share or sharing or collect or collecting or collection or store or storage or storing or access or consent or confidentiality or privacy or security or stewardship or steward or processing or process or custodianship or custody or "secondary use" or "re-use" or "reuse" or "re-analysis" or "reanalyze" or "reanalyse" or "re-analyze" or "re-analyse" or "recontact" or "re-contact" or usage or utilization or utilisation or responsible or responsibility or disclosure) NEAR/5 (genomic or genomics or ((genetic or genetics or gene or genes) NEAR/2 (clinical or test or tests or testing or sequenc* or data or information or predictive or diagnostic or diagnosis or medical)))) | 27090 |
| 6 | TS=(genomic or genomics) | 492777 |
| 7 | TS=((genetic or genetics or gene or genes) NEAR/2 (clinical or test or tests or testing or sequenc* or data or information or predictive or diagnostic or diagnosis or medical)) | 379508 |
| 8 | #6 OR #7 | 821337 |
| 9 | TI=(governance or govern or governing or share or sharing or collect or collection or collecting or store or storage or storing or access or regulation or regulate or regulating or consent or confidentiality or privacy or security or stewardship or steward or processing or process or custodianship or custody or "secondary use" or "re-use" or "reuse" or "re-analysis" or "reanalyze" or "reanalyse" or "re-analyze" or "re-analyse" or "recontact" or "re-contact" or usage or utilization or utilisation or responsible or responsibility or disclosure) OR AK=(governance or govern or governing or share or sharing or store or storage or storing or access or regulation or regulate or regulating or consent or confidentiality or privacy or security or stewardship or steward or processing or process or custodianship or custody or "secondary use" or usage or utilization or utilisation or responsible or responsibility or disclosure) | 4419484 |
| 10 | #8 AND #9 | 41427 |
| 11 | #5 OR #10 | 62982 |
| 12 | TS=(("clinic" or "clinical" or clinician* or "patient" or "patients" or "medicine" or hospital*) and (biobank* or bio-bank* or research* or participant*)) | 1676019 |
| 13 | #11 AND #12 | 4249 |
| 14 | TS=((management or manage or managing or regulation or regulating or regulate or "use") NEAR/2 (genomic* or genetic) NEAR/2 (data or database* or information or registr* or computing or biobank* or bio-bank* or test* or sequenc*)) | 3811 |
| 15 | #14 AND #12 | 595 |
| 16 | #13 OR #15 | 4663 |
| 17 | #4 AND #16 and 2024 or 2023 or 2022 or 2021 or 2003 or 2004 or 2005 or 2006 or 2007 or 2008 or 2009 or 2010 or 2011 or 2012 or 2013 or 2014 or 2015 or 2016 or 2017 or 2018 or 2019 or 2020 (Publication Years) and English (Languages) | 2785 |
| 18 | #4 AND #16 and English (Languages) | 2863 |
| 19 | #4 AND #16 and English (Languages) and 2013 or 2014 or 2015 or 2016 or 2017 or 2018 or 2019 or 2020 or 2021 or 2024 or 2023 or 2022 (Publication Years) | 2316 |

| **Scopus search string, conducted October 10,2024** | **Results** |
| --- | --- |
| ( KEY ( database* OR "genetic testing" OR "genetic predisposition" OR "genetic privacy" OR "privacy" OR "consent" OR "confidentiality" ) OR TITLE ( ( "genetic" OR "genetics" OR "gene" OR "genes" ) W/2 ( "clinical" OR "test" OR "tests" OR "testing" OR sequenc* OR "data" OR "information" OR "predictive" OR "diagnostic" OR "diagnosis" OR "medical" ) ) OR AUTHKEY ( ( "genetic" OR "genetics" OR "gene" OR "genes" ) W/2 ( "clinical" OR "test" OR "tests" OR "testing" OR sequenc* OR "data" OR "information" OR "predictive" OR "diagnostic" OR "diagnosis" OR "medical" ) ) OR TITLE ( "genomic" OR "genomics" OR "data" OR "information" OR biobank* ) OR AUTHKEY ( "genomic" OR "genomics" OR "data" OR "information" OR biobank* ) ) AND ( ( ( ( TITLE-ABS-KEY ( ( governance OR govern OR governing OR share OR sharing OR collect OR collecting OR collection OR store OR storage OR storing OR access OR consent OR confidentiality OR privacy OR security OR stewardship OR steward OR processing OR process OR custodianship OR custody OR "secondary use" OR "re-use" OR "reuse" OR "re-analysis" OR "reanalyze" OR "reanalyse" OR "re-analyze" OR "re-analyse" OR "recontact" OR "re-contact" OR usage OR utilization OR utilisation OR responsible OR responsibility OR disclosure ) W/5 ( genomic OR genomics OR ( ( genetic OR genetics OR gene OR genes ) W/2 ( clinical OR test OR tests OR testing OR sequenc* OR data OR information OR predictive OR diagnostic OR diagnosis OR medical ) ) ) ) ) OR ( ( TITLE-ABS-KEY ( genomic OR genomics ) OR TITLE-ABS-KEY ( ( genetic OR genetics OR gene OR genes ) W/2 ( clinical OR test OR tests OR testing OR sequenc* OR data OR information OR predictive OR diagnostic OR diagnosis OR medical ) ) ) AND ( TITLE ( governance OR govern OR governing OR share OR sharing OR collect OR collecting OR collection OR store OR storage OR storing OR access OR regulation OR regulate OR regulating OR consent OR confidentiality OR privacy OR security OR stewardship OR steward OR processing OR process OR custodianship OR custody OR "secondary use" OR "re-use" OR "reuse" OR "re-analysis" OR "reanalyze" OR "reanalyse" OR "re-analyze" OR "re-analyse" OR "recontact" OR "re-contact" OR usage OR utilization OR utilisation OR responsible OR responsibility OR disclosure ) OR AUTHKEY ( governance OR govern OR governing OR share OR sharing OR collect OR collecting OR collection OR store OR storage OR storing OR access OR regulation OR regulate OR regulating OR consent OR confidentiality OR privacy OR security OR stewardship OR steward OR processing OR process OR custodianship OR custody OR "secondary use" OR "re-use" OR "reuse" OR "re-analysis" OR "reanalyze" OR "reanalyse" OR "re-analyze" OR "re-analyse" OR "recontact" OR "re-contact" OR usage OR utilization OR utilisation OR responsible OR responsibility OR disclosure ) ) ) ) AND ( TITLE-ABS-KEY ( ( "clinic" OR "clinical" OR clinician* OR "patient" OR "patients" OR "medicine" OR hospital* ) AND ( biobank* OR bio-bank* OR research* OR participant* ) ) ) ) OR ( ( TITLE-ABS-KEY ( ( ( {management}Each OR {manage} OR {managing} OR {regulation} OR {regulating} OR {regulate} OR {use} ) W/2 ( {genomic} OR {genomics} OR {genetic} ) ) W/2 ( (70) OR {database} OR {databases} OR {information} OR {registry} OR {registries} OR {computing} OR (71) OR {biobanks} OR {biobanking} OR {bio-bank} OR {bio-banks} OR {bio-banking} OR {test} OR {tests} OR (72) OR {sequence} OR {sequences} OR {sequencing} ) ) ) AND ( TITLE-ABS-KEY ( ( "clinic" OR "clinical" OR clinician* OR "patient" OR "patients" OR "medicine" OR hospital* ) AND ( biobank* OR bio-bank* OR research* OR participant* ) ) ) ) ) AND PUBYEAR > 2012 AND PUBYEAR < 2025 AND ( LIMIT-TO ( LANGUAGE , "English" ) ) | 2813 |
