# Supplementary material for: Genomic Data Governance Challenges Between Research and Clinical Care: A Scoping Review
Source: Health Expect. 2026 Oct 3;29(5):e70889. doi: 10.1111/hex.70889 (PMC13633702; doi:10.1111/hex.70889)
Supplement: Supplementary file 3 [file HEX-29-e70889-s003.docx]

Supplementary material 3. Comprehensive overview of included theoretical/commentary articles (n=25).

| **Reference** | **Jurisdiction** | **Focus** | **Commentary conclusion** | **Cohort-specific** | **Genomic data ELSI considerations** | | | |
| --- | --- | --- | --- | --- | --- | --- | --- | --- |
|  |  |  |  |  | **Lifecycle^[1]^** | **Consent^[2]^** | **Return B/R^[3]^** | **APM^[4]^** |
| (36) | USA | Proposes clinical genomic data elements to be shared by clinical laboratories | Developed points to consider for clinical laboratories on sharing de-identified variant-level data, proposing specific data elements when direct patient consent was not obtained and describing situations in which obtaining informed consent is recommended. | ☐ | ☒ | ☒ | ☐ | ☐ |
| (37) | Canada | describes rare disease data stewardship (from consent to disposal) | With national efforts to integrate whole genomic sequencing into rare disease clinical care, the author recommends that appropriate governance infrastructure, delegation of responsibilities, transparent communication of rights and duties and integrating data privacy safeguards should be established to ensure responsible rare disease data stewardship and sharing. | ☒ | ☒ | ☒ | ☒ | ☐ |
| (38) | USA | proposes the argument for pursuing incidental findings in clinical and research genomic data | Despite its limitations and challenges, the author argues for identifying, studying and returning incidental variants affords opportunities to advance scientific and clinical understanding of genomics. | ☐ | ☒ | ☐ | ☐ | ☐ |
| (39) | USA | describes data sharing among the Undiagnosed Disease network (UDN) | The UDN is developing tools, techniques and best practices to share health and genomic data, aiming to find diagnoses or undiagnosed patients to improve health care and advance scientific understanding. | ☒ | ☒ | ☐ | ☐ | ☐ |
| (6) | EU & USA | the risks and benefits considering the sensitive and digital nature of genomic information | Embedding genomics within digital networks and the Internet creates various privacy and consent challenges that need to be addressed across clinical and research settings. Proposes consent BPs considering IT implications; six recommendations for designing a proactive informed consent process that addresses risks and benefits of digital genomic information Proposes risk management strategies and approaches to data sharing and open access | ☐ | ☐ | ☒ | ☐ | ☐ |
| (40) | USA | proposes principles for building a shared medical info commons | Based on past and present genomic data sharing policies, the authors propose a set of principles on data access and transparency to help achieve a global information commons, aiming to balance open science whilst protecting the rights and interests of contributing individuals. | ☐ | ☒ | ☐ | ☐ | ☐ |
| (41) | USA | explores the case for research participants accessing and sharing raw research genomic data | There are several policy and practice implications of allowing research participants access to raw genomic sequencing data; namely, the (current) lack of obligation of researchers to return results to participants, the quality of research data and results and the lack of funding and resources to plan and appropriately report potentially clinically significant findings | ☐ | ☐ | ☐ | ☒ | ☐ |
| (42) | North America | reviews the importance of (genomic) data reanalysis and sharing for diagnosis among Undiagnosed Rare Disease patients | In describing the challenges of conventional RD diagnostic testing, the authors highlight the importance of recurrent reanalysis of NGS data and sharing data to increase the likelihood of identifying a genetic diagnosis. | ☒ | ☐ | ☐ | ☐ | ☐ |
| (2) | UK | argues for research and clinical practice to co-exist | The authors argue for developing a hybrid governance approach and proposing a re-negotiation of the social contract that recognises the research-clinical interdependent/symbiotic relationship in the context of genomics and acknowledges that research and clinical activities may be a spectrum | ☐ | ☐ | ☐ | ☐ | ☒ |
| (43) | USA | proposes principes for returning research results to participant, minimum and maximum information that should be returned by researchers | Based on their clinical genomics research experience, the authors proposed recommendations for returning genomic test results to research participants. The minimum, the “floor”, that should be returned are research results purposively discovered or by chance that are valid, and medically important and actionable; all genomic information, or the “ceiling”, and everything in-between might be returned depending on if there is an ethical or scientific justification. | ☐ | ☐ | ☐ | ☒ | ☐ |
| (44) | USA | proposes standards to govern research use of clinical genomic data stored in electronic medical records (EMR) | Given the emergence of electronic health records, the authors argue that genomic data should not be treated as de-identified health information and patients should be aware if their data is being disclosed for additional uses such as research. Proposes principles that respects patient confidentiality and autonomy and a model point-of-care notice and disclosure form for EMR-based genomic research. | ☐ | ☒ | ☐ | ☐ | ☐ |
| (45) | N/A | proposes a human rights approach to govern international genomic data sharing | To facilitate global clinical and genomic data sharing, the authors propose a human rights-based international code of conduct as it has universal scope and is legally enforceable, enabling it to regulate behaviour and set norms. | ☐ | ☒ | ☐ | ☐ | ☐ |
| (46) | USA | explores the genomic data policy and practice implications of the 21^st^ Century Cures Act (“the Act”) | While the Act promotes a favourable environment for an Information Commons, the authors note that it also reinforces the siloing of research and clinical care. | ☐ | ☒ | ☐ | ☐ | ☐ |
| (47) | USA | highlights the ELSI policy issues associated with genomic data in implementing a learning health system | Despite all three countries taking different approaches to implement precision medicine and genomic testing into their respective healthcare systems, the authors discuss the ethical and policy considerations that they believe will need to be addressed to realise these implementation efforts. The authors propose that a new ‘social license’ is required to conduct research in the precision medicine era is required to ensure its conduct and impact align with what is socially desirable, fair and appropriate. | ☐ | ☐ | ☐ | ☐ | ☒ |
| (48) | GA4GH | proposes principles for responsible paediatric genomic data sharing | By considering the ethical, legal and social implications of paediatric data sharing, the GA4GH’s Interdisciplinary Paediatric Task Team proposed four policy domains intended to maximise benefits for the patient’s family: children’s involvement; parental consent; balancing benefits and risks; and data protection and release. The authors contend that enabling access to paediatric data benefits current and future patients and that those who generate such data have a duty to provide children and their families the opportunity to share that data. | ☐ | ☒ | ☐ | ☐ | ☐ |
| (7) | GA4GH | describes GA4GH and their work to facilitate genomic data sharing via standards and frameworks | Presents the strategic goals of the GA4GH and details its strategies, approaches and vision to enable responsible genomic and clinical data sharing to advance genomic medicine and research | ☐ | ☒ | ☐ | ☐ | ☐ |
| (49) | USA | describes the privacy and confidentiality risks associated with genomic databases | While the authors acknowledge that large (National Human Genome Research Institute) genomic databases will generate collaboration and discoveries, they also highlight that these databases can also create risk to privacy and confidentiality. The authors encourage that all individuals should inform and consult their families and be counselled before participating in genomic testing to ensure all relevant people are informed on these risks. | ☐ | ☒ | ☒ | ☐ | ☐ |
| (50) | Belgium | explores the ELSI implications of (genomic) data collected using non-traditional methods | Non-traditional data collection approaches for biomedical research purposes (e.g. mobile collected data) creates complexities regarding the nature of data processing and the legal grounds and requirements for processing data as individuals may not be aware of the secondary uses of their data. | ☐ | ☒ | ☒ | ☐ | ☐ |
| (51) | N/A | explores the ethical issues associated with genomic data and testing across research, clinical and individual use | The use of genetic tests presents ethical issues that need to be considered to ensure it is appropriately and responsibly applied. The author debated the different ethical conflicts and considerations arising from using genetic testing in clinical practice, research and direct-to-consumer/individual use, concluding that informed consent is fundamentally important in genetic testing. | ☐ | ☒ | ☒ | ☐ | ☐ |
| (52) | EU | considers the ethical and legal challenges associated with genomics, describes ‘best practices’ addressing these issues often through legislation | This policy brief highlights how legislation and regulation offer best practice to address the ethical and legal challenges associated with genomic data. In consultation with all stakeholders, effective regulation and legislation such as the 2018 European Union General Data Protection Regulation (GDPR) shapes how genomic data are managed and processed across settings. | ☐ | ☒ | ☐ | ☐ | ☐ |
| (53) | Canada; EU | examines the ethical and legal debate in precision cancer medicine and translational sciences | In addition to addressing the various ethical and legal issues, the authors believe that a major societal shift is required to implement precision medicine and genomics into health care. | ☐ | ☐ | ☐ | ☐ | ☒ |
| (54) | USA | proposes a translational approach to ethics for genomics | Using genomics as an example, the authors propose an ethical framework for translational science by describing a method for resolving conflict between different ethical frameworks across the stages of the translation cycle. | ☐ | ☐ | ☐ | ☐ | ☒ |
| (55) | USA | proposes principles to address legal conflicts between competing legal frameworks | The authors present a translational approach to the law of genomics, providing recommendations and a strategy for resolving conflicts between different settings’ legal frameworks: prioritise the protection of the rights and interest of the most vulnerable – individuals undergoing genomic testing and providing their data. | ☐ | ☐ | ☐ | ☐ | ☒ |
| (56) | Netherlands | identifies a combined research-care approach to address the core needs of a learning health system | By offering an approach to combine care and research, learning health care may provide solutions for some of precision medicine’s central needs. Various ethical factors need to be considered, namely consent, oversight and independent review and public accountability and engagement. | ☐ | ☒ | ☒ | ☐ | ☒ |
| (57) | UK | proposes ‘best practices’ for addressing the potential identification of misattributed parentage | Using a case study of identifying misattributed parentage through research genomic testing, the authors recommend that the possibility of finding and discussing parentage should be addressed during the pretest counselling and consent process and that clinical relevance should determine whether or not to disclose results in the clinical setting. | ☐ | ☒ | ☒ | ☐ | ☐ |

1. Specific data lifecycle stage
2. Consent considerations
3. Return of benefit and results
4. Advancing precision medicine
